# A new common functional coding variant at the DDC gene change renal enzyme activity and modify renal dopamine function

**DOI:** 10.1038/s41598-019-41504-7

**Published:** 2019-03-25

**Authors:** Jose Pablo Miramontes-Gonzalez, Makena Hightower, Kuixing Zhang, Hiroki Kurosaki, Andrew J. Schork, Nilima Biswas, Sucheta Vaingankar, Manjula Mahata, Michael S. Lipkowitz, Caroline M. Nievergelt, Dewleen G. Baker, Michael G. Ziegler, David León-Jiménez, Rogelio González-Sarmiento, Hiroshi Ichinose, Daniel T. O’Connor

**Affiliations:** 1 Departments of Medicine, Pharmacology, Psychiatry, and Institute for Genomic Medicine, University of California at San Diego, La Jolla, CA USA; 2 Department of Life Science, Graduate School of Bioscience and Biotechnology, Tokyo Institute of Technology, Yokohama, Japan; 3 VA San Diego Healthcare System, Department of Medicine, La Jolla, CA USA; 4 Georgetown University, Washington, DC USA; 5 Hospital Universitario de Salamanca, Instituto de Investigación Biomedical de Salamanca (IBSAL), Salamanca, Spain

## Abstract

The intra-renal dopamine (DA) system is highly expressed in the proximal tubule and contributes to Na+ and blood pressure homeostasis, as well as to the development of nephropathy. In the kidney, the enzyme DOPA Decarboxylase (DDC) originating from the circulation. We used a twin/family study design, followed by polymorphism association analysis at *DDC* locus to elucidate heritable influences on renal DA production. Dense single nucleotide polymorphism (SNP) genotyping across the *DDC* locus on chromosome 7p12 was analyzed by re-sequencing guided by trait-associated genetic markers to discover the responsible genetic variation. We also characterized kinetics of the expressed DDC mutant enzyme. Systematic polymorphism screening across the 15-Exon DDC locus revealed a single coding variant in Exon-14 that was associated with DA excretion and multiple other renal traits indicating pleiotropy. When expressed and characterized in eukaryotic cells, the 462Gln variant displayed lower Vmax (maximal rate of product formation by an enzyme) (21.3 versus 44.9 nmol/min/mg) and lower Km (substrate concentration at which half-maximal product formation is achieved by an enzyme.)(36.2 versus 46.8 μM) than the wild-type (Arg462) allele. The highly heritable DA excretion trait is substantially influenced by a previously uncharacterized common coding variant (Arg462Gln) at the DDC gene that affects multiple renal tubular and glomerular traits, and predicts accelerated functional decline in chronic kidney disease.

## Introduction

Dopamine (DA) in the central and peripheral neural systems has an established role in motor and behavior control. The kidney also possesses a dopaminergic system that seems to be independent from neural DA systems. In fact intra-renal production of DA is not regulated by renal sympathetic nerve activity as evidenced by renal denervation^1^. Instead, DA is formed locally in proximal tubule epithelial cells^2^ from its circulatory precursor levodopa (L-DOPA) after filtration at the glomerulus. L-DOPA is transported into tubular cells, where it is decarboxylated to DA by the enzyme DOPA Decarboxylase (DDC), which is regulated by dietary sodium (Na+)^3,4^. DA then exits these cells across apical and basolateral surfaces to exert paracrine actions via G-protein coupled DA receptors, predominantly dopamine receptor1(DRD1) across the nephron, signaling largely through G_s_ to adenylyl cyclase^5^. The renal dopaminergic system is thus a physiological regulator of Na+ excretion through inhibition of tubular Na+ reabsorption.

Dopaminergic actions in the kidney are not limited to maintaining Na+ homeostasis. DA may increase glomerular filtration rate (GFR) by post-glomerular (efferent) arteriolar constriction^6^ and modulates renin expression^7^ as well as angiotensin II, also controlling Na+ excretion and blood pressure (BP)^8,9^.

Not surprisingly, defects in renal DA production or receptor signaling are linked to development of hypertension^10–12^ In animal models intra-renal DA production is linked to development of hypertension and decreased longevity^13^, as well as angiotensin II-mediated renal injury^14^ and progression of nephropathy^15^.

We used a classical twin design approach to ask whether renal DA synthesis and excretion are heritable, and we performed a genetic association analysis to determine specific effects of the *DDC* locus on the urinary DA trait. Finally, we have performed a functional analysis of 462Gln DDC variant.

## Methods

All protocols were approved by the appropriate committees of the University of California – San Diego, Human Research Protection Program (HRPP), and each subject gave written informed consent prior to participation.

### Subjects and clinical characterization

#### Twin and sibling subjects

The University of California, San Diego (UCSD) twin/sibling study has previously been described^16^. Twin and sibling participants were recruited from southern California by access to a population birth record-based twin registry^17^, as well as by newspaper advertisement as described previously^18^. Subjects included both dizygotic (DZ) and monozygotic (MZ) twin pairs. Zygosity of twins was confirmed genetically by microsatellite and SNP markers^18^. Clinical characterization was conducted as previously described^18^. BP status (high vs. normal) was defined by history (medical record or self-report), presence of antihypertensive medications, and measurement of seated BP by arm cuff [hypertension: either/or ≥140/≥90 mmHg systolic BP (SBP)/diastolic BP (DBP), or both]. None of the subjects had a history of cardiovascular disease or renal failure, and plasma creatinine concentrations were ≤1.5 mg/dL. Estimated glomerular filtration rate (eGFR) was estimated by the MDRD (Modification of Diet in Renal Disease) algorithm.

#### Twin/sibling physiological phenotyping

Brachial cuff BPs with SBP/DBP measured as K1/K4, as well as heart rate, were obtained non-invasively in seated subjects in triplicate using an oscillometric device (DynaPulse; PulseMetric Inc., Vista, CA) as described^16^. Triplicate values (within ±10% of the mean) were averaged. We and others previously validated DynaPulse measurements against more invasive devices^16,19^.

#### Twin/sibling biochemical phenotyping

Subjects were instructed to fast during the 6 h preceding the evaluation. Fasting blood and freshly voided urine samples were collected from each subject. Plasma and urine samples were quickly frozen to −70 °C, in preparation for later catecholamine assay at the same time. A sensitive radioenzymatic assay was used to measure plasma and urine catecholamines [dopamine (DA), norepinephrine (NE), and epinephrine (EPI)] through the catechol-O-methylation process^20^. The radioenzymatic assay for catecholamines involved transfer of a ^3^H label to catecholamines from S-adenosylmethionine during O-methylation, mediated by the enzyme catechol-O-methyltransferase (COMT). Prior to O-methylation, plasma catecholamines were extracted into dilute acetic acid to remove COMT inhibitors in plasma. Assay sensitivities (lower limits of detection) were ~10 pg for DA and NE, and ~6 pg for EPI. Inter-assay coefficients of variation were ~10% for urine DA, ~20% for plasma DA, ~10% for NE and ~13% for EPI. Plasma and urine creatinine were measured by autoanalyzer (Beckman-Coulter; Brea, CA), and urine catecholamine results were normalized to creatinine in the same sample.

### Genotyping of the DDC locus

We obtained the exon/intron structure for human *DDC*, as well as putative sequence, at http://genome.ucsc.edu, focusing on the longer kidney/liver (~107 kbp) isoform (RefSeq NM_001082971.1).

Genomic deoxyribonucleic acid (DNA) from twin and sibling pairs was isolated from blood leukocytes with Qiagen columns, after proteinase K digestion, as previously described^18^. SNP genotyping across the *DDC* locus was accomplished using the Illumina 610-Quad genotyping array www.Illumina.com. *DDC* LD blocks are displayed in Supplementary Information Fig. 1.

#### Systematic polymorphism characterization across the *DDC* locus

After initial positive *DDC*-on-DA excretion association we searched for functional variants across the *DDC* locus by re-sequencing exons, untranslated regions (UTRs), and promoter, in 12 subjects who carried the trait-associated (minor) allele in intron-8 tagging SNP rs11575358 (G > A), versus 11 subjects who did not carry this allele. A public draft of the human^21^ genome sequence was obtained from the UCSC Genome Bioinformatics website http://genome.ucsc.edu and used as a scaffold for primer design and sequence alignment. Positions were numbered with respect to the messenger ribonucleic acid (mRNA) cap (transcriptional initiation) site in national Center for Biotechnology Reference Sequences (NCBI RefSeq) source clone NM_001082971.1 (kidney and liver splicing isoform), using polymerase chain reaction (PCR) primers designed by Primer3^22^ to span each of the 15 exons, and 50–100 bp of flanking intronic sequences, as well as 965 bp of the proximal promoter (upstream of the cap site). Primers are shown in Supplementary Table 1. PCR, amplicon purification and analysis of target sequences were performed according to the protocol described previously^23^. Sequence was determined on an ABI-3730XL dideoxy capillary sequencer. Polymorphism and heterozygosity were visualized from the ABI tracings using Codon Code Aligner http://www.codoncode.com/aligner.

#### Genotyping of coding variant Arg462Gln (rs11575542, G/A) in *DDC* exon-14

An amplified fragment length polymorphism (AFLP) analysis was also used to genotype the Arg462Gln at the DDC locus, with forward primer 5′-ggcttcttcctgatgtacgg-3′, and reverse primer 5′-aataaggaagagaaggccgg-3′. Following PCR (30 plus cycles) and digestion with restriction enzyme StuI (1 unit, 2 hours), 2% agarose gel electrophoresis separated restriction fragments. Genotyping yielded a single 482-bp fragment for G/G (Arg/Arg, major allele) homozygotes; two shorter fragments (257 and 225 bp) for A/A homozygotes (Gln/Gln); while G/A heterozygotes (Arg/Gln) displayed 3 fragments (482, 257, and 225 bp) (Supplemental Fig. 2).

### Replication of marker (*DDC*)-on-trait (DA) association

#### MariMRSnes

US Marines (all young healthy men) from the Marine Resiliency Study (MRS) http://marineresilience.org/^24^ were evaluated. Of 2600 Marines assessed at pre-deployment for MRS on bases in Southern California, 361 were included in this analysis. Participants were healthy males of self-identified European (white, non-Hispanic) ancestry, ranging in age from 18–37 years (mean 21.7 ± 2.5 years). Biological samples including freshly voided urine, nucleated cells from whole blood (for genomic DNA) and EDTA plasma by venipuncture were collected for further analysis.

#### Caregivers

The study population included caregivers from The Alzheimer Caregiver Coping Study^25^, conducted at UCSD. To be eligible for the study, participants were required to be at least 55 years of age, married, and providing in-home care for their spouses. Participants were excluded if they were diagnosed with a serious medical condition, had hypertension greater than 200/120 mmHg, or if they were taking anti-coagulant medication (exclusion criteria due to other data collected for this prospective study).

### Statistical analyses

#### Heritability (h^2^) and genetic covariance

Heritability (h^2^) estimates of physiological and biochemical traits were obtained through twin pair (MZ or DZ) variance components in Sequential Oligogenic Linkage Analysis Routines (SOLAR)^26^ available at http://solar.txbiomedgenetics.org. Heritability is estimated by the equation: h^2^ = V_G_/V_P_, where V_G_ is additive genetic variance and V_P_ is total phenotypic variance. Here a normal distribution in twin pairs is assumed in order to maximize the likelihood with a mean dependent on traits of interest. Genetic covariance of DA excretion with other correlated heritable traits was estimated as ρ_G_ in SOLAR, while environmental covariance was estimated as parameter ρ_E_.

SNP-on-phenotype effects across the *DDC* locus were initially tested in MERLIN v1.1.2 http://www.sph.umich.edu/csg/abecasis/merlin/ with an additive model, to explicitly account for family structure. Descriptive (mean ± SEM) and inferential statistics were computed by generalized estimating equations (GEEs) to account for correlated observations within families, using SPSS (IBM Corporation; Armonk, NY) predictive analytics software. GEE also tested for allele and diploid genotype effects on specified traits. The SNP was defined as a 3-level variable representing the three possible genotypes (homozygous variant, heterozygous, and homozygous wild-type), initially testing an additive effect in the model. Analyses were adjusted for age and sex. In addition to additive models, after inspection of distribution of the observations, heterozygotes and minor (or major) allele homozygotes were grouped together, as needed to test dominant/recessive models.

#### Marker-on-trait association display

Results across the DDC locus, centered on intronic tagging-SNP peak associations (e.g., rs11575340) as well as results centering on functional variant Arg462Gln (rs11575542) were displayed by local SNAP (SNP Annotation and Proxy Search) plot as described at http://www.broadinstitute.org/mpg/snap/ldplot.php.

Grouped analysis were carried out testing fixed-effect (i.e., diploid genotype) models in STATA (v12, Stata Corp, College Station, TX), after individual study regression analyses in SPSS (v17), incorporating individual data to derive significance as well as pooled genotype effect size (beta, or slope/allele) and its SE (standard error).

#### Bioinformatics

To determine conservation of sequence at the functional coding genetic variant (Arg462Gln) and local homology, we performed sequence searches using BLASTP http://blast.ncbi.nlm.nih.gov, followed by multiple sequence alignments with CLUSTAL Omega (1.1.0) http://www.ebi.ac.uk/Tools/msa/clustalo/.

### Human DDC enzyme (EC 4.1.1.28) coding genetic variant Arg462Gln (rs11575542): Mutagenesis, eukaryotic expression, and characterization of enzymatic kinetics

#### Site-directed mutagenesis

The full-length human *DDC* cDNA DNA clone, was obtained from a human pheochromocytoma cDNA library, and code for a 480 amino acid protein with a predicted molecular mass of ~53.9 kDa. The cDNA was inserted (in the correct 5′ → 3′ orientation) into a eukaryotic (pSV40 promoter) expression plasmid, as previously described^13^. The wild-type (Arg462) version was used as a template for site-directed mutagenesis (QuikChange, Agilent) to create the 462Gln variant, which was verified by dideoxy-sequencing.

#### COS cell transfection and electrophoresis

COS cells were transfected with the pSV40 *DDC* expression plasmids with a modified calcium-phosphate procedure as described^13^, followed by cell lysis, SDS-PAGE electrophoresis, protein transfer to polyvinylidene difluoride membranes (Bio-Rad, Hercules, CA), and either protein staining (coomassie blue) or detection by immunoblotting with a rabbit anti-human DDC polyclonal antibody, to visualize the previously described M_r_ ~ 50 kDa immunoreactive band. We also detected beta-actin with an anti-beta-actin monoclonal antibody (Sigma A5441, St. Louis, MO) to normalize the expression level of DDC protein in COS cells, with protein loads of 25, 50, and 100 µg protein/lane (in triplicate).

#### Expressed human DDC enzyme (EC 4.1.1.28) kinetics: Vmax and Km

COS-cell-expressed DDC (Arg462 versus Gln462) gave rise to enzymatic activity that catalyzed the decarboxylation of L-3,4-dihydroxyphenylalanine (L-DOPA) to DA, at pH 6.5 in the presence of cofactor pyridoxal phosphate (10 µM). Cell lysates were prepared and enzymatic activity (including Km (substrate concentration at which half-maximal product formation is achieved by an enzyme) as a function of substrate concentration, and Vmax (maximal rate of product formation by an enzyme) at saturating substrate concentration). Km and Vmax values were calculated using a Hanes-Woolf plot. Vmax results were normalized to DDC protein expression, determined by immunoblot DDC/beta-actin ratio (in triplicate).

After transfection of COS cells with DDC-expression plasmid, we performed a Western blotting to estimate the expression levels of wild type (WT) or variant DDC protein. The expression level of variant DDC was 83% less than that of WT DDC (as shows in the results), probably caused by the differences in transfection efficiency and the copy number of expression plasmids in the cells. We assayed the DDC activity in the same lysates of transfected COS cells, and determined Km and Vmax values of each lysate. Then, the Vmax value of variant DDC was divided by 0.83 to normalize with the expression level of WT DDC.

## Results

### Renal DA excretion: Heritability (h^2^) and genetic covariance

**Figure 1:**
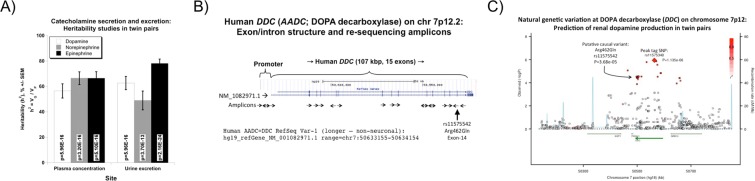
Renal DA excretion: Effects of heredity and the *DDC* gene. (**A**) Heritability (h^2^) of catecholamine secretion and urinary excretion. Results for plasma catecholamine concentrations (pg/ml) and urine catecholamine excretion (ng/gm creatinine) emerge from variance components analyses by SOLAR in MZ versus DZ twin pairs. h^2^ is expressed as % of trait variance attributable to gene action, ±SEM. V_G_: genetic variance; V_P_: total phenotypic variance. (**B**) Human DOPA decarboxylase (DDC): Exon/intron and inter-species sequence conservation. Polymorphism discovery across the human *DDC* locus: re-sequencing strategy. There are 15 exons at human *DDC*. Eighteen polymerase chain reaction amplicons spanned the promoter and coding regions. Amplicons with double arrows were read in both directions. The SNP Arg462Gln (rs11575542) characterized in this paper is located in exon-14. (**C**) Natural genetic variation at DOPA decarboxylase (*DDC*, *AADC*) on chromosome 7p12: Prediction of renal DA production in twin pairs. Local region of the Manhattan plot displayed by SNAP (SNP Annotation and Proxy Search) plot http://www.broadinstitute.org/mpg/snap/ldplot.php. The peak association (p = 1.35E-06; see diamond) was at *DDC* intron-5 tagging SNP rs11575340. The DA secretion trait-associated coding variant (characterized in this paper) is at *DDC* Arg462Gln (exon-14, rs11575542). Marker-on-marker LD is shown as R^2^ (with respect to rs11575340), on a red color scale.

Twin pairs variance component analyses indicated that catecholamine secretion and renal excretion traits were all substantially heritable (Table 1, Fig. 1A), with DA excretion heritability (h^2^ = V_G_/V_P_) = 62.7 ± 5.2% of trait variance (p = 5.96E-16). Notably, the renal and plasma DA traits did not significantly correlate, nor did they display shared heritability (genetic covariance), reinforcing the separate control of the two systems.

**Table 1:** Heritability h^2^ and genetic covariance: Shared genetic and environmental co-determination for traits correlated with urinary dopamine excretion.

Trait	Correlation with uDopamine/creat		Trait heritability (h^2^)			Rho_E (environmental covariance)			Rho_G (genetic covariance)			n (individuals)
	Spearman Rho	P-value	Estimate	SEM	P-value	Estimate	SEM	P-value	Estimate	SEM	p	
**Urine catecholamine**												
uDA/creat	—	—	**0**.**627**	**0**.**052**	**5**.**96E-16**	—	—	—	—	—	—	—
uNE/creat	**0**.**381**	**1**.**74E-12**	**0**.**490**	**0**.**074**	**1**.**00E-07**	**0**.**264**	**0**.**089**	**4**.**70E-03**	**0**.**612**	**0**.**089**	**6**.**33E-08**	325
uEpi/creat	**0**.**530**	**2**.**44E-24**	**0**.**781**	**0**.**035**	**2**.**16E-24**	**0**.**548**	**0**.**068**	**1**.**07E-09**	**0**.**541**	**0**.**068**	**1**.**71E-09**	325
**Plasma catecholamine**												
pDA	0.104	6.83E-02	**0**.**565**	**0**.**056**	**1**.**82E-13**	**0**.**294**	**0**.**086**	**1**.**48E-03**	−0.020	0.108	8.52E-01	346
pNE	−0.017	7.62E-01	**0**.**652**	**0**.**050**	**3**.**19E-16**	−0.061	0.092	5.09E-01	0.043	0.105	6.78E-01	355
pEPI	**−0**.**120**	**3**.**33E-02**	**0**.**665**	**0**.**051**	**5**.**09E-16**	0.068	0.092	4.57E-01	−0.066	0.107	5.40E-01	353
**Renal**												
eGFR (MDRD)	**0**.**166**	**2**.**94E-03**	**0**.**662**	**0**.**049**	**3**.**59E-17**	**0**.**193**	**0**.**088**	**3**.**34E-02**	**0**.**266**	**0**.**098**	**1**.**01E-02**	347
uAlbumin/creat	**0**.**234**	**2**.**35E-05**	**0**.**357**	**0**.**085**	**7**.**56E-05**	0.175	0.091	5.93E-02	0.200	0.136	1.54E-01	369
FELi^+^	**0**.**269**	**2**.**02E-05**	**0**.**390**	**0**.**083**	**3**.**06E-05**	**0**.**237**	**0**.**096**	**1**.**88E-02**	**0**.**296**	**0**.**109**	**1**.**12E-02**	341
FENa^+^	**0**.**216**	**2**.**08E-04**	**0**.**321**	**0**.**083**	**2**.**29E-04**	**0**.**269**	**0**.**091**	**5**.**32E-03**	0.210	0.134	1.38E-01	347

Covariance estimates (±SEM) are from analyses in SOLAR. Heritability, shared genetic determination (genetic covariance, ρ_G_ also known as pleiotropy) and environmental determination (environmental covariance, ρ_E_) for traits correlated with urine DA excretion are indicated. ρ_G_ and ρ_E_ are fractions, scaled from −1 to 0, and 0 to +1, as determined in SOLAR. Spearman non-parametric trait-on-trait correlations (Rho) are also reported. MDRD, algorithm for eGFR. Significant differences (*p* < 0.05) are bold. Analyses were undertaken in MZ and DZ twin pairs of European ancestry.

### Renal DA excretion: *Cis*-QTL at the *DDC* locus

During dense SNP genotyping across the *DDC* locus (Fig. 1B), we observed a broad *DDC* marker-on-DA trait association peak in twin/sibling pairs (Fig. 1C) spanning the ~107 kbp *DDC* locus, but confined to a ~275 kbp linkage disequilibrium (LD) block at the locus, as defined by the local recombination pattern (cM/Mb tracing). A peak tagging SNP (rs11575340, within intron-5) was highly associated with urine DA excretion levels (p = 1.135E-06). By contrast, the plasma DA trait did not display association with the *DDC* locus (all p > 0.05). Other *DDC* SNPs “tagging” the urinary DA trait at high significance (p < 1.0E-05) were rs11575358 (intron-8), rs11575522 (intron-12), and exonic SNP Arg462Gln (rs11575542, exon-14). The association of *DDC* with DA excretion was confirmed with additional independent groups (Twins/siblings, Marines, and caregivers) using tagging SNPs rs11575358 and rs11575522. Grouped analyses indicate significant associations for each marker SNP rs11575358 (p = 1.0E-04 OR: 0,879), and rs11575522 (p = 1.0E-04 OR: 0,890) (Table 2).

**Table 2:** Replication of the effects of DOPA decarboxylase (DDC) tagging variants on renal dopamine excretion: Grouped analysis of 3 independent population groups.

Group	DDC variant	Alleles, Minor/Major	Minor allele frequency	N	Trait	Beta (slope per allele)	SE (of beta)	P-value	OR	95% CI
Twins/siblings	rs11575385	T/C	3.48%	453	Urine DA/creat	−0.173	0.030			
Marines/MRS	rs11575385	A/G	1.72%	228	Urine DA/creat	0.080	0.064			
Caregivers	rs11575385	A/G	0.68%	132	Urine DA/creat	−0.234	0.317			
**Result**	**rs11575385**	—	—	**813**	**Urine DA/creat**	**−0**.**129**	**0**.**027**	**<0**.**0001**	**0**,**879**	(**0**,**834**;**0**,**927**)
Twins/siblings	rs11575522	T/C	2.90%	453	Urine DA/creat	−0.16	0.031			
Marines/MRS	rs11575522	A/G	2.16%	229	Urine DA/creat	0.040	0.060			
Caregivers	rs11575522	A/G	1.37%	132	Urine DA/creat	−0.1034	0.226			
**Result**	**rs11575522**	—	—	**813**	**Urine DA/creat**	**−0**.**116**	**0**.**028**	**<0**.**0001**	**0**,**890**	(**0**,**843**;**0**,**941**)

Effects of *DDC* tagging variants rs115575385 (28 kb from peak tagging SNP for urine DA excretion, rs11575340) and rs11575522 (61 kb from peak tag SNP rs11575340) on urine DA excretion in three independent groups, analyzed with STATA. MRS indicates Marine Resiliency Study; DA/creat, DA excretion normalized to creatinine in the same urine sample. Significant (p < 0.05) effects are given in bold type. During analyses, tests of potential heterogeneity of samples were positive for both rs11575386 (Q statistic = 12.9, df = 2, p = 0.002) and rs11575522 (Q = 8.85, df = 2, p = 0.012).

The trait-associated *DDC* region on chromosome 7p12, was also plotted in CEU subjects at www.HapMap.org as pair-wise LD parameter R^2^ across the ∼107 kbp locus (Supplemental Fig. 1), indicating a substantial LD block spanning the locus.

### Trait aggregation with DA excretion: Stratification by urine DA quantiles

We next asked whether other traits were associated with DA excretion. Table 3 provides a description of the twin/sibling cohort, divided into two groups (quantiles, upper and lower) stratified around the DA excretion median value. Elevated urine DA was substantially more common in women than men (p = 1.5E-07), a trend borne out by direct comparison of excretion in women versus men (173.1 ± 3.68 versus 134.6 ± 5.49 ng/gm, p = 1.0E-5). Participants with higher DA excretion showed significant (*p* < 0.05) elevations in several other traits, including urine catecholamines (NE and EPI), excretion of K+, Na+, and Cl−, as well as the fractional excretion of Na^+^ (FENa^+^) (though not fractional excretion of Li+. (FELi+)), and eGFR.

**Table 3:** Urine dopamine quantiles: Effects on demographic, biochemical and physiological traits.

Traits	Urine dopamine quantiles (median)						P-value
	Lower: <153.8 µg/g			Upper: •153.8 µg/g			
	n	Mean	SEM	n	Mean	SEM	
**Demographic and Physical**							
Age, years	185	40.3	1.2	188	39.4	1.2	
Sex: 1 = M, 2 = F (M/F)	185	66/119		188	19/169		**3**.**00E-05**
BP status, NT/HTN (%)	185	171/14 (92%/8%)		188	168/20 (89%/11%)		2.95E-01
BMI, kg/m^2^	184	24.7	0.31	184	24.8	0.31	7.57E-01
**Biochemical**							
Catecholamines							
Urine dopamine, µg/g	185	121.9	3.35	185	206.1	3.33	**<1**.**0E-06**
Urine norepinephrine, µg/g	185	24.7	0.75	182	31.6	0.76	**<1**.**0E-06**
Urine epinephrine, µg/g	182	10.8	0.42	182	14.8	0.41	**<1**.**0E-06**
Plasma dopamine, pg/ml	173	19.9	2.07	176	21.9	2.05	5.20E-02
Plasma norepinephrine, pg/ml	175	307.2	11.1	175	332.5	11.1	2.32E-01
Plasma epinephrine, pg/ml	178	25.2	1.23	176	26.6	1.24	5.60E-01
Renin–angiotensin system							
Plasma renin, pg/ml	176	19.6	1.18	182	19.3	1.16	9.97E-01
Plasma aldosterone, pg/ml	177	139.2	5.59	178	127.9	5.57	1.94E-01
Inflammation							
C reactive protein, ng/ml	167	1648.6	184.0	178	2117.0	178.0	4.40E-01
Kidney and urine							
Urine albumin excretion, mg/g	181	8.87	1.93	186	10.31	1.91	6.27E-01
eGFR (MDRD), ml/min	178	87.8	1.61	181	96.2	1.60	**9**.**00E-03**
Urine K+, mEq/g	185	64.3	2.44	186	73.4	2.43	**1**.**90E-02**
Urine Na+, mEq/g	185	115.9	5.12	186	150.9	5.12	**<1**.**0E-06**
Urine Cl−, mEq/g	184	135.2	5.36	183	170.1	5.32	**<1**.**0E-06**
Urine cortisol, μg/g	132	4.34	0.25	145	3.78	0.24	2.75E-01
Urine nitric oxide, nmol/g	177	1035.7	54.2	181	1213.8	53.6	8.70E-02
FELi+, %	120	24.8	4.44	140	24.1	4.09	4.47E-01
FENa+, %	161	1.59	0.19	163	2.23	0.19	**1**.**90E-02**
**Physiological**							
Hemodynamics							
Systolic BP, mmHg	170	122.6	0.99	177	123.1	0.86	5.19E-01
Diastolic BP, mmHg	171	76.3	0.64	178	93.7	0.83	4.55E-01
Heart rate, beats/min	178	60.1	0.79	182	69.9	0.86	4.55E-01

Results for twin/sibling study population, divided about the median value for urine DA excretion. Descriptive (mean ± SEM) and inferential statistics (p-values) for twins/siblings were derived from generalized estimating equations (GEE) to account for correlations within nuclear families. Numbers (n) describes the number of individuals analyzed. BP indicates blood pressure; NT, normotensive; HTN, hypertensive; CRP, C reactive protein; eGFR MDRD, estimated GFR; FELi+, fractional excretion of lithium; FENa+, fractional excretion of Na+; NO, nitric oxide; DBP, diastolic blood pressure; SBP, systolic blood pressure; HR, heart rate; BMI, body mass index; SV resistance, systemic vascular resistance. Significant differences (P <0.05) are in bold type.

However, subjects with increased DA excretion did *not* display changes in circulating catecholamines (including plasma DA) or components of the renin-angiotensin-aldosterone system), BP or heart rate (HR).

Visual display of trait aggregation for excreted DA (p = 1.0E-06) with NE (p = 1.0E-06) and EPI, (p = 1.0E-06), is given in Supplemental Fig. 3a. Supplemental Figure 3b displays the significant correspondence of urine DA with urine Na^+^ excretion (p = 1.0E-06) and FENa^+^: increasing levels of excreted Na^+^ led to higher FENa^+^ (p = 0.019). Supplemental Figure 3c displays urine DA and eGFR coordinately increasing (p = 9.0E-03). The effect on glomerular function is reflected in the excretion of other electrolytes: K+ (p = 0.019) and Cl− (p = 1.0E-06) (Table 3).

### Systematic polymorphism discovery across the *DDC* locus

In 23 unrelated individuals (n = 46 chromosomes) stratified by presence/absence of *DDC* rs11575358 trait-associated (“risk”, A, minor) allele (11 with, 12 without), the promoter and all 15 exons and adjacent intronic regions were re-sequenced. The 17 amplicons and their sequencing directions are represented in Fig. 1B. As shown in Table 4 a total of 17 polymorphisms were detected. Of these, two common variants were discovered in the promoter: a common SNP (rs56233242, G-899T) and an Insertion/Deletion (Ins/Del) variant (rs76759613, G-554/−). We identified 2 non-synonymous (amino acid replacement) SNPs, one previously known and located in exon-2 (E2-206; G/A, Val60Met) and a previously uncharacterized SNP located in exon-14 (rs11575542, E14-143; G/A, Arg462Gln). We also found a second novel SNP in intron-3 (I3-21; C/T) and a common five-base Ins/Del in intron-9 (rs59827423, I9-107; CAGGG/−). The other 10 SNPs detected were common and intronic.

**Table 4:** Summary of systematic DOPA decarboxylase (*DDC*) polymorphism determination by re-sequencing across the locus: Discovery of trait-associated coding variant Arg462Gln.

Genetic variant			Allele			Codon					Diploid genotypes		Statistics
SNP#	Position −/+ cap site	RefSNP_#	Major	Minor	SNP location	Codon-1	Codon-2	Codon #	Amino acid_1	Amino acid_2	“Controls” (G_allele at tagging rs11575385; N = 12)	“Cases” (A_allele at tagging rs11575385; N = 11)	Fisher Exact Test P-value (case versus control)
1	−899	rs56233242	G	T	Promoter						6_G/G, 4_T/G, 2_T/T	2_G/G, 3_T/G, 6_T/T	0.26635
2	−554	rs76759613	G	—	Promoter						3_G/G, 7_G/−, 2_−/−	7_G/G, 4_G/−	0.13980
3	E2-206	New	G	A	Exon_2	GTT	ATT	60	Val	Ile	11_G/G, 1_G/A	11_G/G	1.00000
4	I2-37	rs11575293	A	G	Intron_2						12_A/A	2_A/A, 8_G/A, 1_G/G	**0**.**00007**
5	E3-33	rs11575302	C	T	Exon_3	GCC	GCT	78	Ala	Ala	12_C/C	1_CC, 9_C/T, 1_T/T	**0**.**00003**
6	I3-21	New	C	T	Intron_3						12_C/C	10_CC, 1_T/C	0.47826
7	I4-16	rs11575334	T	C	Intron_5						2_T/T, 6_T/C, 4_C/C	4_T/T, 6_T/C, 1_C/C	0.35444
8	I5-42	rs3735273	A	G	Intron_5						5_G/G, 6_A/G, 1_A/A	2_G/G, 5_AG, 4_A/A	0.23631
9	I6-22	rs11575375	T	C	Intron_7						6_C/C, 4_T/C, 2_T/T,	6_T/C, 5_T/T	0.14045
10	I8-84	rs11575392	A	G	Intron_8						6_A/A, 5_A/G, 1_G/G	8_A/A, 2_A/G, 1_G/G	0.40032
11	I9-63	rs4947580	G	A	Intron_9						12_G/G	10_G/G, 1_G/A	0.23913
12	I9-107	rs59827423	CAGG	—	Intron_10						3_CAGGG/CAGGG, 5_CAGGG/−, 2_−/− (two missing)	6_CAGGG/−, 5_−/−	0.38128
13	I9-23	rs11575457	T	G	Intron_10						4_T/T, 6_T/G, 2_G/G	4_T/G, 7_G/G	**0**.**01100**
14	I11-9	rs11575482	G	A	Intron_11						6_G/G, 1_A/A, 5_A/G,	7_G/G, 4_A/G,	1.00000
15	E14-143	rs11575542	G	A	Exon_14	CGG	CAG	462	Arg	Gln	12_G/G	1_GG, 9_G/A, 1_A/A	**0**.**00001**
16	I14-42	rs11575543	C	T	Intron_14						12 _C/C	8_C/C, 2_C/T, 1 T/T	0.09317
17	E15-323	rs11575551	C	T	Intron_15						9_C/C, 3_C/T	7_C/C, 4_C/T	0.66685

The location for each polymorphism is given, and their positions are numbered upstream (−) or downstream (+) of the cap (transcription initiation) site. For each SNP, the reference number (RefSNP) is given where available in the public database, and novel polymorphisms are indicate as “New”. Nucleotide deletion is indicated by “−”. The Fisher Exact Test P values compare individuals carrying/not carrying the risk (A, minor) allele at tagging intronic polymorphism rs11575385 (G > A).

We selected 23 subjects based on genotype, according to trait-associated intron-8 tagging SNP rs11575358 (G > A): 11 cases (carrying the DA-trait-associated A-allele, one A/A homozygote and 10 G/A heterozygotes) and 12 controls (G/G homozygotes).

Inter-species amino acid sequence alignments and conservation are presented in Supplemental Table 2. Results reveal that Arg 462 is completely in old world primates, though not in new world primates (squirrel monkey, marmoset). 462Gln is found in several other mammalian and vertebrate species: mole rat, guinea pig, and ricefish. The local region around codon 462 is within a carboxy-terminal alpha-helix (helix-12, amino acids 457–477) on X-ray crystallography of human DDC.

### Pleiotropic effects of *DDC* genetic variant Arg462Gln on multiple human traits

With diploid genotypic ratios of Arg/Arg:Arg/Gln:Gln/Gln at 429:23:1, Arg462Gln (rs11575542) was in Hardy Weinberg equilibrium (HWE) (Chi^2^ = 1.32, p = 0.25), with a minor allele frequency of ~2.76%. Its effects on physiological and biochemical traits were evaluated in twins/siblings (Table 5), revealing pleiotropic effects of Arg462Gln on multiple traits.

**Table 5:** Genetic marker-on-trait associations between DDC coding variant Arg462Gln (rs11575542, G > A) and phenotypes in twins and siblings.

Traits	Arg462Gln (G>A) diploid genotype						P-value
	Arg/Arg (G/G)			Gln/Arg (G/A) or Gln/Gln (A/A)			
	n	Mean	SEM	n	Mean	SEM	
**Demographic and Physical**							
Age, years	429	40.1	1.41	24	38.1	2.3	3.57E-01
Sex: 1 = M, 2 = F	110/31	—	—	18-Jun	—	—	9.44E-01
BP status, NT/HTN	384/45	—	—	23/1	—	—	3.18E-01
BMI, kg/m^2^	423	24.9	0.224	24	26.2	1.1	1.29E-01
**Biochemical**							
Catecholamines							
Urine dopamine, μg/g	429	150.3	2.52	24	208.3	10.3	**3.68E-05**
Urine norepinephrine, μg/g	423	28.2	0.576	23	26.5	2.13	4.40E-01
Urine epinephrine, μg/g	419	10.9	0.237	24	15.7	1.21	**1.06E-03**
Plasma dopamine, pg/ml	401	11.4	0.017	22	10.6	0.073	6.67E-01
Plasma norepinephrine, pg/ml	403	325.5	6.99	23	276.4	29.3	1.44E-01
Plasma epinephrine, pg/ml	405	21.1	0.663	23	25.9	3.32	4.16E-01
Renin-angiotensin system							
Plasma renin, pg/ml	340	19.6	0.831	18	16.6	3.61	3.62E-01
Plasma aldosterone, pg/ml	377	134.1	3.7	19	110.5	16.3	3.28E-01
Inflammation							
C reactive protein, ng/ml	389	2.93	0.029	23	3.03	0.121	3.24E-01
Kidney and urine							
Urine albumin excretion, mg/g	439	4.76	0.24	23	6.79	1.55	**3.30E-02**
eGFR (MDRD), ml/min	411	94.6	1.22	22	110.5	6.81	**3.20E-02**
Urine K+, mEq/g	425	70.3	1.5	23	68.3	6.6	5.01E-01
Urine Na+, mEq/g	426	129.4	3.34	23	162.9	14.3	**1.20E-02**
Urine Cl−, mEq/g	424	149.9	3.5	23	175.1	14.9	5.49E-01
Urine cortisol, ug/g	265	3.31	0.11	12	2.52	0.23	6.96E-01
Urine nitric oxide, nmol/g	412	932.8	342	23	1213.5	344.9	1.46E-01
FELi+, %	248	13.9	0.603	11	23.1	3.2	**8.00E-03**
FENa+, %	372	1.79	0.127	18	3.64	0.575	**4.70E-02**
**Physiological**							
Hemodynamic							
Systolic BP, mmHg	399	123.2	0.63	21	128	2.57	7.33E-01
Diastolic BP, mmHg	401	75.8	0.46	21	79.1	2.11	7.05E-01
Heart rate, beats/min	412	69.1	0.51	23	74.5	2.17	4.76E-01

Numbers (n) describe number of individuals analyzed for that genotype and trait. BP indicates blood pressure; NT, normotensive; HTN, hypertensive; CRP, C reactive protein; eGFR, estimated GFR; FELi+, fractional excretion of Li+; FENa+, fractional excretion of Na+; NO, nitric oxide; DBP, diastolic blood pressure; SBP, systolic blood pressure; HR, heart rate; BMI, body mass index. Entries reflect mean ± one SEM. Significant (p < 0.05) differences are indicated in bold type. Descriptive statistics emerged from GEE. Inferential statistics (p-values) emerged from MERLIN, except for FELi+ and FENa+ (from GEE) and urine albumin excretion (by non-parametric Kruskal-Wallis test).

**Figure 2:**
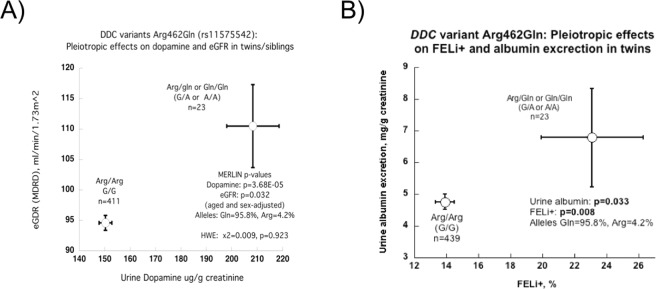
Association of *DDC* genetic variant Arg462Gln (rs11575542) with DA excretion and renal function. Results are shown as mean ± SEM in twins/siblings. Numbers are in parentheses (n) indicate the n for the observation. Significant differences (p < 0.05) are in bold. (**A**) Pleiotropic effects on urine DA excretion, p = 3.68E-05, and eGFR, p = 0.032. Minor allele homozygotes Gln/Gln (A/A) and heterozygous Arg/Gln (G/A) subjects displayed increased eGFR and urine DA excretion compared to homozygous major allele Arg/Arg (G/G) subjects. (**B**) Pleiotropic effects on FELi+ (p = 0.008) and urine albumin excretion (p = 0.033). Minor allele homozygotes (Gln/Gln, A/A) and heterozygotes (Arg/Gln, G/A) displayed increased FELi+, as well as urine albumin excretion, compared to major allele homozygotes (Arg/Arg, G/G).

Notable effects were observed in the renal excretion traits. Carriers of the 462Gln (minor) allele (heterozygous [n = 23] or homozygous [n = 1]) showed increased DA and EPI excretion. Directionally coordinate effects on eGFR and urine DA are depicted in Fig. 2A. Figure 2B displays parallel effects on albumin excretion and FELi^+^, an index of proximal tubular avidity for Na^+^. Thus 462Gln-augmented renal DA synthesis and excretion may give rise to a cascade of events in the kidney: local DA may trigger both natriuresis and afferent arteriolar vasodilation (or efferent constriction), and the ensuing glomerular hyperfiltration increases albumin excretion.

Notably, *DDC* variant Arg462Gln did *not* affect plasma catecholamine (including plasma DA) concentrations, the renin-angiotensin system (plasma renin and plasma aldosterone), c-reactive protein (CRP) levels, or systemic hemodynamic traits (BP and HR) (Table 5).

### Human DDC variant enzyme kinetics

#### Expression

**Figure 3:**
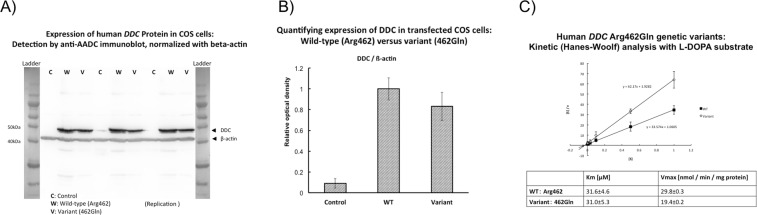
Human DDC enzyme genetic variants. (**A**) Expression of human *DDC* protein in COS cells: Detection by anti-AADC immunoblot, normalized with beta-actin. Protein standard size ladders bracket the experimental results. C (control), W (wild-type, Arg462), and V (variant, 462Gln). Blots were initially examined at protein loads of 25, 50, and 100 µg protein/lane; here, triplicate determinations for 50 µg protein loads are shown, along with size standards. (**B**) Quantifying expression of DDC expressed in COS cells: Wild-type (Arg462) versus variant (462Gln). DDC and beta-actin immunoreactive bands (at 50 µg protein/lane, in triplicate) were scanned for optical density, and mean ± one SD values are shown. Expression of 462Gln was ~83% of that for Arg462. (**C**) Kinetic analyses with L-DOPA substrate. Graph shows a Hanes-Woolf plot of [S]/v as a function of [S], where [S] is the concentration of substrate [L-DOPA] and v is the reaction velocity (nmol/min/mg) at that [S]. The 462Gln variant displays a substantially lower Vmax (at 19.4 ± 0.2 versus wild-type 29.8 ± 0.3 nmol/min/mg protein; 35% reduction at p < 0.0001), though similar Km (at 31.0 ± 5.3 vs wild-type 31.6 ± 4.6 μM; p = 0.94). Replicates: at [S] = 0.5, n = 2; others, n = 3. Results are shown as mean ± one SD. Since typical L-DOPA substrate concentrations in humans are far below the Km value (at L-DOPA of ~1.08 nM [2.13 ng/ml] in plasma and ~100 nM [1.22 µg/hr] in urine, then the DDC system is likely to be catalyzing DA synthesis over a relatively linear range *in vivo*.

Figure 3A shows immunoblot results of human *DDC* protein expression in COS cells: wild-type (Arg462) and variant (Gln462), with beta-actin control normalization. Figure 3B shows the results of the quantifying the expression of DDC in transfected cos cells in controls (column 1), wild type (Arg462) and variant (462Gln), with a higher expression in wild type cells.

#### Kinetics

We analyzed the kinetic parameters comparing wild-type and variant enzymatic action using L-DOPA as substrate (Fig. 3C), using a Hanes-Woolf plot. The 462Gln variant displayed a *substantially* lower Vmax (at 19.4 ± 0.2 versus wild-type 29.8 ± 0.3 nmol/min/mg protein; 35% reduction at p < 0.0001), though similar Km (at 31.0 ± 5.3 versus wild-type 31.6 ± 4.6 μM).

## Discussion

The independent renal DA system highly expressed in proximal tubule subserves both tubular and glomerular functions. Intra-renal DA also influences the renin-angiotensin system and thereby controls progression of renal damage in kidney pathologies^14,15^.

The role of common genetic variation in control of endogenous dopaminergic activity is previously unexplored. In the twin study, we first determined that DA excretion is a highly heritable trait (at 62.7 ± 5.2% of trait variance, p = 5.96E-16), although it was genetically uncorrelated with plasma DA. We then characterize a functional non-synonymous variant (Arg462Gln) in the *DDC* coding region that seems to control common heritable variation in DA secretion and its downstream renal consequences in an apparently healthy population. Replication was performed with a grouped analysis of three independent cohorts that validated the findings (Table 2).

### The paradox of elevated DA excretion in *DDC* deficiency

We observed a *DDC* genetic variant (Arg462Gln) wherein the minor allele (462Gln) seemed to increase renal DA production but reduce enzymatic DDC activity. Mendelian human autosomal recessive DDC deficiency is characterized by predominantly neurological manifestations such as developmental delay, prominent motor abnormalities, oculogyric crises and autonomic features^27,28^. Available treatment options (DA agonists, vitamin B6 and monoamine oxidase inhibitors) are only variably effective^29^. However, many such *DDC*-deficient patients exhibit normal or even *elevated* levels of *urinary* DA excretion in the presence of a less active DDC with normal levels of plasma DA^30^. Such findings have been referred to the “paradox of hyperdopaminuria in *DDC* deficiency”. Clues to the origin of this paradox involve the presence of a separate/local renal DA system synthesizing the transmitter in proximal tubular epithelial cells of the kidney cortex^31^. This local kidney system displays high efficiency in transforming blood-derived L-DOPA to renal DA. As a consequence of systemic DDC deficiency in the brain and elsewhere, more substrate (L-DOPA) and cofactors may be delivered to DDC in the kidney cortex^32^. Other theories propose alternative substrates for renal DA formation such as 3-methoxy-tyrosine^33^, other metabolic pathways such as tyramine hydroxylation by renal CYP2D6 to form DA^34^ or even tyrosinase as an alternative pathway for DOPA and DA synthesis even in the absence of tyrosine hydroxylase^35^.

### Selective kidney *DDC* loss-of-function in experimental animals: Renal functional consequences

The *DDC* 462Gln allele elevate DA eGFR and albumin excretion in healthy individuals (Table 5, Fig. 2B). These results suggest that glomerular hyperfiltration may be a risk factor for accelerated renal decline in 462Gln carriers^36^. By contrast, after selective loss of intra-renal DA production by targeted ablation of the *DDC* gene in the proximal tubule, transgenic mice display *protection* from renal injury induced by angiotensin II^14^ or during diabetic nephropathy^15^.

Why might humans and mice display such apparent differences in renal effects of intra-renal DA deficiency? First of all, in the mouse, *DDC* ablation could be created precisely in the proximal tubule by targeting the knockout to cells expressing the *Cre* recombinase driven by a proximal tubule-specific (γ-GT) promoter^37^, while humans express *DDC* from the same gene in both CNS and kidney. Secondly, our results as well as other human studies on the “paradox of hyperdopaminuria in DDC deficiency” indicate that a relatively *inactive* DDC enzyme can actually result in *elevated* renal DA excretion^32^ by several proposed mechanisms. Thus, the human and mouse results may ultimately be compatible.

### Advantages and limitations of this study

Here we were able to mobilize several complementary techniques to understand genetic control of renal dopamine excretion: twin pair variance components to estimate heritability, extensive human phenotyping, dense SNP genotyping, validation by grouped analysis of three independent groups, systematic polymorphism discovery across the *DDC* locus, functional testing of a mutant enzyme, and extension into a disease population. The non-synonymous amino acid substitution (Arg462Gln) occurred in a carboxy-terminal alpha-helix far from the DDC active site^38^; however, the Arg → Gln substitution yields a substantial change in amino acid properties specified by codon 462 (cationic → neutral), and its position far from the active site is consistent with the unchanged Km (i.e., no change in substrate affinity) in the face of diminished Vmax (i.e., essentially non-competitive or allosteric inhibition).

Nonetheless, questions remain. DDC and DA production are widespread, occurring not just in the kidney, but also in liver and in the nervous system. In the intact human organism (as opposed to a more manipulable experimental animal) it may be difficult to understand precisely the roles of such different sites of origin of the amine. Indeed, there may be other origins of DA, such as tyramine hydroxylation by renal CYP2D6^34^ or even tysosinase^39^. Since there is only a single *DDC* gene on chromosome 7p12, subserving expression in both brain and kidney (albeit with alternative initial exons), and since DDC is in the biosynthetic pathway for serotonin as well as dopamine, it is likely that our results also have implications for neuropsychiatric disease; however, in this report we confined our investigation to renal traits.

### Conclusions and perspectives

**Figure 4:**
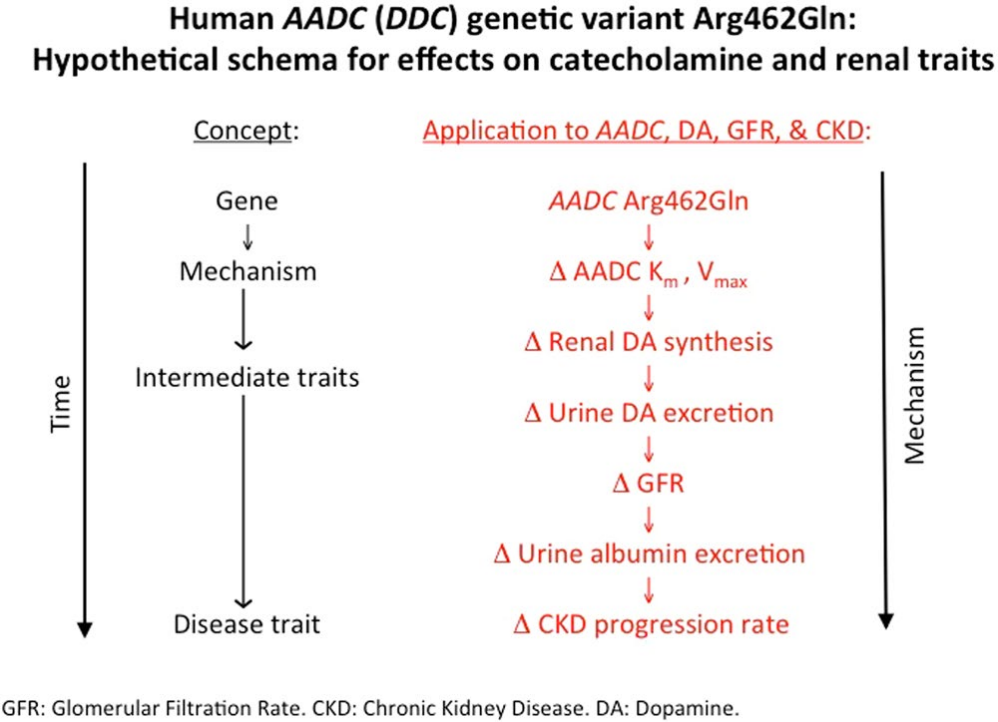
Hypothetical schema for effects of *DDC* genetic variation on catecholamine and renal traits. The genetic variation of human CDD and its effects on AD and renal traits: a schematic hypothesis. This framework correlates the experimental results from the genetic variation of the gene that codes for CDD, the change in the activity of the CDD enzyme and how it influences the first DA excretion (biochemical trait), then affects the excretion of electrolytes and GFR (traits). physiological), the excretion of albumin in urine and, finally, determines the progression of CKD (disease trait).

We conclude that *DDC* exon-14 non-synonymous variant Arg462Gln is functional and this variant has clinical implications as follows, and it alters enzyme kinetic parameters, Vmax Km, resulting in changes in urine DA excretion. The change in DA secretion influences renal function, modifying renal tubular avidity for Na+ and Li+, with consequent changes in eGFR and albumin excretion, all this changes eventuating in accelerated renal decline in patients with CKD (Fig. 4). These findings augment our understanding of the kidney dopaminergic system, and shed new light on the link between DA production and renal dysfunction in humans, a topic previously investigated largely in animal models.

## Supplementary information

Suplementary Info.
