# Author Correction: A new common functional coding variant at the DDC gene change renal enzyme activity and modify renal dopamine function

**DOI:** 10.1038/s41598-020-59785-8

**Published:** 2020-02-14

**Authors:** Jose Pablo Miramontes-Gonzalez, C. Makena Hightower, Kuixing Zhang, Hiroki Kurosaki, Andrew J. Schork, Nilima Biswas, Sucheta Vaingankar, Manjula Mahata, Michael S. Lipkowitz, Caroline M. Nievergelt, Dewleen G. Baker, Michael G. Ziegler, David León-Jiménez, Rogelio González-Sarmiento, Hiroshi Ichinose, Daniel T. O’Connor

**Affiliations:** 1 Departments of Medicine, Pharmacology, Psychiatry, and Institute for Genomic Medicine, University of California at San Diego, La Jolla, CA USA; 2 Department of Life Science, Graduate School of Bioscience and Biotechnology, Tokyo Institute of Technology, Yokohama, Japan; 3 VA San Diego Healthcare System, Department of Medicine, La Jolla, CA USA; 4 Georgetown University, Washington, DC USA; 5 Hospital Universitario de Salamanca, Instituto de Investigación Biomedical de Salamanca (IBSAL), Salamanca, Spain

Correction to: *Scientific Reports* 10.1038/s41598-019-41504-7, published online 25 March 2019

The original version of this Article contained a typographical error in the spelling of the author C. Makena Hightower, which was incorrectly given as Makena Hightower. This has now been corrected in the PDF and HTML versions of this article, and in the accompanying Supplementary Information file.

As a result, the Author Contribution section,

“J.P.M.G., M.S.L., M.G.Z., D.G.B., M.M., D.T.O. and H.I.: designed the project. J.P.M., M.H., K.Z., S.V., N.B., M.M. and A.J.S.: Performed the genetic experiments. H.K. and H.I.: performed the enzyme kinetics experiments. J.P.M.G., K.Z. and M.H. wrote the main manuscript text. J.P.M.G., D.T.O., C.M.N., D.L.-J., R.G.S. and D.G.B., reviewed the manuscript.”

now reads:

“J.P.M.G., M.S.L., M.G.Z., D.G.B., M.M., D.T.O. and H.I.: designed the project. J.P.M., C.M.H., K.Z., S.V., N.B., M.M. and A.J.S.: Performed the genetic experiments. H.K. and H.I.: performed the enzyme kinetics experiments. J.P.M.G., K.Z. and C.M.H. wrote the main manuscript text. J.P.M.G., D.T.O., C.M.N., D.L.-J., R.G.S. and D.G.B., reviewed the manuscript.”

