# Supplementary material for: A new common functional coding variant at the DDC gene change renal enzyme activity and modify renal dopamine function
Source: Sci Rep. 2019 Mar 25;9:5055. doi: 10.1038/s41598-019-41504-7 (PMC6433864; doi:10.1038/s41598-019-41504-7)
Supplement: Supplementary file 1 — Suplementary Info. [file 41598_2019_41504_MOESM1_ESM.pdf]

A new common functional coding variant at the DDC gene change renal enzyme activity and modify renal dopamine function

Jose Pablo Miramontes-Gonzalez MD/PhD[1,3,5], Makena Hightower PhD[1], Kuixing Zhang MD/PhD[1,3], Hiroki Kurosaki BS[2], Andrew J. Schork PhD[1] Nilima Biswas PhD[1,3], Sucheta Vaingankar[1,3], Manjula Mahata PhD[1,3], Michael S. Lipkowitz MD[4], Caroline M. Nievergelt PhD[1], Dewleen G. Baker MD[1,3], Michael G. Ziegler MD[1], David León Jiménez MD[2], Rogelio González Sarmiento MD PhD[1,5], Hiroshi Ichinose PhD[2], Daniel T. O'Connor MD[1,3*].

From

1.Departments of Medicine, Pharmacology, Psychiatry, and Institute for Genomic Medicine, University of California at San Diego, La Jolla, CA, USA.

2.Department of Life Science, Graduate School of Bioscience and Biotechnology, Tokyo Institute of Technology, Yokohama, Japan.

3.VA San Diego Healthcare System, La Jolla, CA. Department of Medicine,

4.Georgetown University, Washington, DC. USA

5.Hospital Universitario de Salamanca. Instituto de Investigación Biomedical de Salamanca (IBSAL). Salamanca, Spain.

*Professor Danniel T. O`Connor: Deceased

Key words: Dopamine, DOPA, DOPA decarboxylase, twin, heredity.

Address for correspondence:

J. Pablo Miramontes González, jpmiramontes@hotmail.com.

Internal Medicine Unit. Hospital Universitario de Salamanca. Instituto de Investigación Biomedical de Salamanca (IBSAL).

SUPPLEMENTAL FIGURES AND LEGENDS.

Supplemental Figure 1. Haplotype blocks across the *DDC* locus: Results from HapMap. LD marker-on-marker map and haplotype blocks were constructed from CEU (European ancestry) data at <www.HapMap.org>. The color scheme (white → pink → red) represents increasing value of LD, with maximum D’ in bright red.

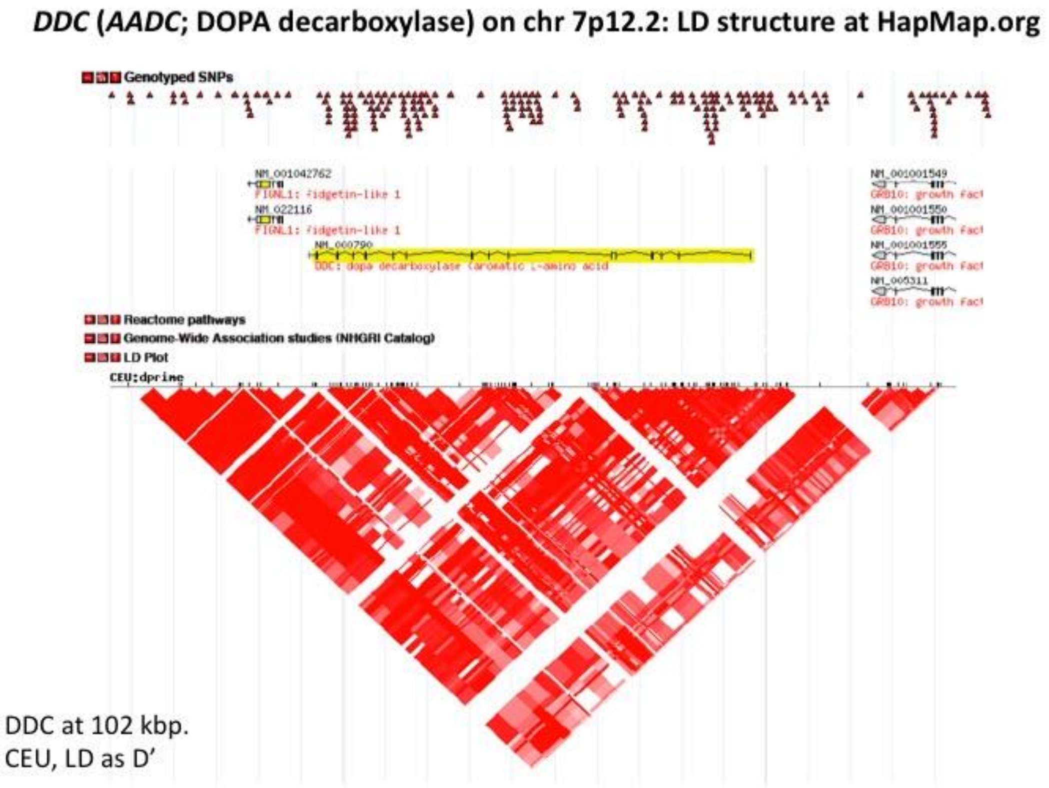

Supplemental Figure 2. AFLP analysis of *DDC* variant Arg462Gln (rs11575542, G>A) . G/G: 482 bp; G/A: 482, 257, 225 bp; A/A: 257, 225 bp.

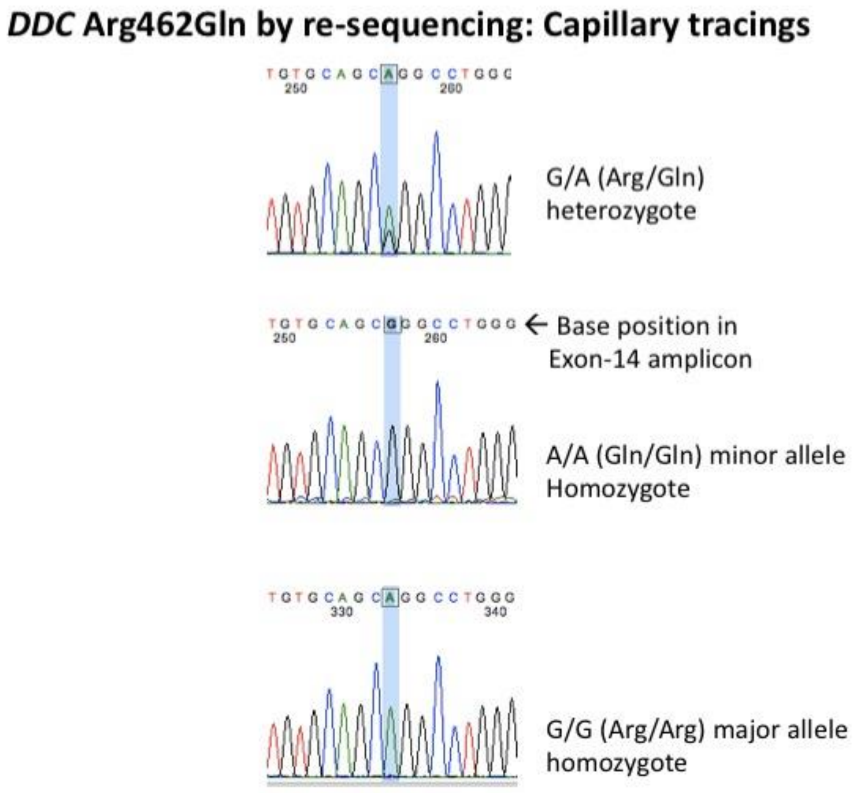

Supplemental Figure 3. Trait aggregation of urine DA (uDA) excretion with biochemical and physiological phenotypes. Results in upper and lower uDA quantiles (shown as mean ± SEM) were analyzed by GEE in twins/siblings. Numbers are in parentheses (n) indicate the n for the observation. Significant differences ($p<0.05$) are **bold**.

(3a). Effects on the urine excretion of all three catecholamines: DA (uDA), p=1.0E-06; NE (uNE), p=1.0E-06; and EPI (uEpi), p=1.0E-06.

(3b). Joint effects on urine Na+ excretion, p=1.9E-02, and FENa+, p=1.0E-06.

(3c). Joint effects on urine DA excretion (uDA), p=1.0E-06, and eGFR, p=9.0E-03.

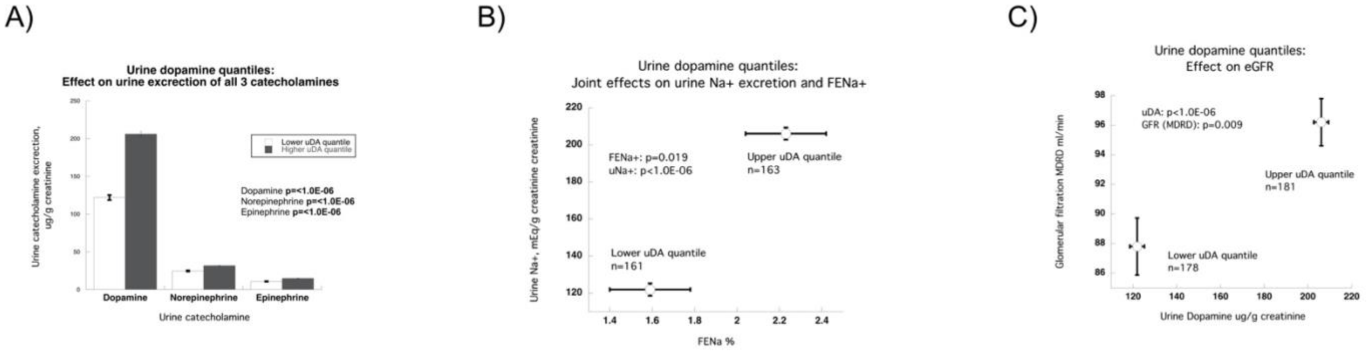

Supplemental Tables

Supporting Table 1: Replication of the effects of DOPA decarboxylase (DDC) tagging variants on renal dopamine excretion: Grouped analysis of 3 independent population groups. Effects of *DDC* tagging variants rs115575385 (28 kb from peak tagging SNP for urine DA excretion, rs11575340) and rs11575522 (61 kb from peak tag SNP rs11575340) on urine DA excretion in three independent groups, analyzed with STATA. MRS indicates Marine Resiliency Study; DA/creat, DA excretion normalized to creatinine in the same urine sample. Significant (p<0.05) effects are given in **bold** type. During analyses, tests of potential heterogeneity of samples were positive for both rs11575386 (Q statistic =12.9, df=2, p=0.002) and rs11575522 (Q=8.85, df=2, p=0.012).

Group	DDC variant	Alleles, Minor/Major	Minor allele frequency	N	Trait	Beta (slope per allele)	SE (of beta)	P-value
Twins/siblings	rs11575385	T/C	3.48%	453	Urine DA/creat	-0.173	0.030	
Marines/MRS	rs11575385	A/G	1.72%	228	Urine DA/creat	0.080	0.064	
Caregivers	rs11575385	A/G	0.68%	132	Urine DA/creat	-0.234	0.317	
**Result**	**rs11575385**	**-**	**-**	**813**	**Urine DA/creat**	**-0.129**	**0.027**	**<0.0001**
Twins/siblings	rs11575522	T/C	2.90%	453	Urine DA/creat	-0.16	0.031	
Marines/MRS	rs11575522	A/G	2.16%	229	Urine DA/creat	0.040	0.060	
Caregivers	rs11575522	A/G	1.37%	132	Urine DA/creat	-0.1034	0.226	
**Result**	Rs11575522	-	-	**813**	**Urine DA/creat**	-0.116	0,028	**<0.0001**

Table 2: *DDC* variant Arg462Gln conservation across species. The Arg462Gln position is indicated by **red/bold type**. Species versions were identified by sequence homology at BLASTP <http://blast.ncbi.nlm.nih.gov>. Multiple sequence alignment was performed by Clustal Omega (1.1.0). Conservation legend: * = fully conserved; : = conservation between groups of strongly similar properties (scoring >0.5 in Gonnet PAM 250 matrix), . = conservation between groups of weakly similar properties (scoring <0.5 in Gonnet PAM 250 matrix); blank = not conserved.

**Primer number**	**Orientation**	**Sequence (5'→3')**
DDC P1	Sense	ccctcactcttccccttacc
	Anti-sense	cccaaacccatcaaaatcat
DDC P2	Sense	gccagaaagaccagctccta
	Anti-sense	tctgggaatcctgagagcac
DDC E1	Sense	tgggctccaaacttgaaatc
	Anti-sense	accacacaagctccctcaag
DDC E2	Sense	tcctacagacatggagggaaa
	Anti-sense	tgccatagggattccttgaa
DDC E3	Sense	aatgggatggctgacatttg
	Anti-sense	ctgcaacagtagcccgtct
DDC E4	Sense	ttcaggcctttgaatcacatc
	Anti-sense	ccaagagctcggttttgttt
DDC E5	Sense	ggacacaaaacaatatgtcttcca
	Anti-sense	cacccttccctgtagttca
DDC E6	Sense	ggagagggagcagtcaggag
	Anti-sense	tacaagaatgcgccaccat
DDC E7	Sense	catccatgggcttacgtttc
	Anti-sense	tctgagtttgtggagttcaagc
DDC E8	Sense	tttggggattcagccattag
	Anti-sense	aaggctctcagcccctattc
DDC E9	Sense	ccagcactccagaagactcc
	Anti-sense	ggttcagaaaaggcagcaag
DDC E10	Sense	cccaggtacttggagcagag
	Anti-sense	tacaagggcaaatccaggaa
DDC E11	Sense	ctcgggacacacccttagaa
	Anti-sense	gactgaggggttcagcagag
DDC E12	Sense	aagaaagcctctgcccacat
	Anti-sense	tgggggacaagagtgaaact
DDC E13	Sense	cttagcctgcctggaaacag
	Anti-sense	aaagaatgcaggctttgctc
DDC E14	Sense	ggcttcttcctgatgtacgg
	Anti-sense	ccggccttctcttccttatt
DDC E15	Sense	gagagggagagggacttcttta
	Anti-sense	tgccgtttaaaaacatccaa
